# Adaptive Fixed-Time Control of Strict-Feedback High-Order Nonlinear Systems

**DOI:** 10.3390/e23080963

**Published:** 2021-07-27

**Authors:** Yang Li, Jianhua Zhang, Xiaoyun Ye, Cheng Siong Chin

**Affiliations:** 1 School of Information and Control Engineering, Qingdao University of Technology, Qingdao 266525, China; jianhuazhang@qut.edu.cn (J.Z.); yexiaoyun@qut.edu.cn (X.Y.); 2 Faculty of Science, Agriculture, and Engineering, Newcastle University Singapore, Singapore 599493, Singapore; cheng.chin@ncl.ac.uk

**Keywords:** adaptive fixed-time control, neural network control, strict-feedback high-order nonlinear systems

## Abstract

This paper examines the adaptive control of high-order nonlinear systems with strict-feedback form. An adaptive fixed-time control scheme is designed for nonlinear systems with unknown uncertainties. In the design process of a backstepping controller, the Lyapunov function, an effective controller, and adaptive law are constructed. Combined with the fixed-time Lyapunov stability criterion, it is proved that the proposed control scheme can ensure the stability of the error system in finite time, and the convergence time is independent of the initial condition. Finally, simulation results verify the effectiveness of the proposed control strategy.

## 1. Introduction

Recently, the adaptive trajectory tracking control of uncertain nonlinear systems has made a significant breakthrough [1,2,3]. In addition, neural network adaptive control has become a popular method in the past decades [4,5,6]. Many remarkable results have extended to strict-feedback systems, pure-feedback systems, and Brunovsky systems, and neural networks are combined with various techniques, such as the backstepping technique, the adaptive technique, and the sliding mode control method [7,8,9]. The neural network is used to identify the nonlinear term of the uncertain system, which combines the advantages of adaptive control. Many excellent articles and monographs have been published. In the design of these control systems, the neural network is used as a general approximator to the uncertain nonlinear term of the systems [10,11,12]. In these systems, the unknown nonlinear systems are approximate by neural networks, which are valid only within a compact set, and the neural network controller is designed. Based on Lyapunov uniformly bounded (UUB) theory, the closed-loop error systems are bounded [13,14,15]. In order to overcome the problem of uncertainty or disturbance that does not meet the specific matching conditions, the adaptive controller is usually constructed by combining backstepping control technology with the adaptive neural network. The high-order system is divided into multiple subsystems. The virtual controller of the low-order subsystem is designed first. Then, the recursive design is used until the final design of the neural network adaptive controller to achieve stability of the system, allowing it to possess the desired performance indicators.

In practical engineering applications, the research of high-order nonlinear systems has attracted much attention, and their application is also extensive, for example, as financial systems, communication systems, biological systems, and machine systems [16,17,18]. Some results regarding high-order system control have been obtained following the development of adding a power integrator [19]. The problems studied in recent years involve robust control [20,21], adaptive global stabilization [17], global asymptotic stabilization [22], output feedback stabilization [23], and state feedback output tracking [16]. Many methods have been proposed, such as backstepping technology, adaptive technology, sliding mode control, neural network control, and fuzzy control. However, the above results need to be precise with some unknown coefficients in the system model. In [20], the unknown function in the system is described by the mathematical model of an online neural network. In addition to this pioneering result, high-order system control based on neural networks has been widely developed and applied [24,25,26].

In the actual industrial process, such as in missile systems, aircraft attitude control systems, robot control systems and other industrial control systems, the purpose of controller design is to achieve stability of the controlled system and maintain it for a limited time. However, the control method without considering the convergence time cannot achieve this objective. Compared with the traditional Lyapunov stability theory, the finite-time Lyapunov stability theory has attracted the attention of many researchers because it can make the controlled system stable near the equilibrium state in finite time [27,28,29].

Many researchers combine finite-time control with neural network adaptive control for nonlinear systems with nonlinear functions and dynamic uncertainties based on backstepping and propose many related adaptive finite-time control schemes [30,31,32]. However, there are still many problems to be solved in these existing control strategies. For finite-time control, the convergence time is dependent on the initial condition. However, the ideal weights of NNs are unknown, and it is difficult to obtain a convergence time. Therefore, to solve this issue, fixed-time neural network control is an appropriate selection of the control method.

The high-order systems’ neural network control problem is discussed in the articles [33,34,35]. The fixed-time neural network adaptive controller is present for nonlinear high-order systems. Based on the fixed-time adaptive technology, the strict-feedback high-order system has fixed-time Lyapunov stability based on Lyapunov stability theory [36,37,38]. The convergence time of the system can be accurately calculated, and the settling time does not rely on the initial situation. The main contributions of this article are as follows:

(1) The combination of the neural network adaptive control with fixed-time Lyapunov stability theory for high-order nonlinear system control problems.
(2) The design of the fixed-time adaptive law of the error systems for neural networks. The parameters of neural networks are iteratively in fixed time based on the Lyapunov fixed-time stability theorem.
(3) The convergence time set by control parameters and adaptive law gain parameters without initial conditions to ensure the control performance.

This article consists of the following sections: in Section 2, a strict-feedback high-order nonlinear mathematical description of the problem is presented; in Section 3, the adaptive fixed-time neural network control scheme for the strict-feedback high-order nonlinear system is designed; in Section 4, simulation results show the effectiveness of the proposed control strategy; in Section 5, the conclusion of the article is presented.

## 2. Problem Formation and Preliminaries

Consider the following strict-feedback high-order nonlinear system:

(1) $$\begin{array}{l} {\dot{x}}_{i}=g_{i}x_{i+1}^{\eta_{i}}+f_{i}\left({\bar{x}}_{i}\right)\\ {\dot{x}}_{n}=g_{n}u^{\eta_{n}}+f_{n}\left({\bar{x}}_{n}\right)\\ y=x_{1} \end{array}$$

where $x_{i}\in R$ is the state of the system; ${\bar{x}}_{i}={\left[x_{1},\ldots ,x_{i}\right]}^{T}\in R^{i}$ is the state vector of the system; $f_{i}\left({\bar{x}}_{i}\right):R^{i}\to R$ is the unknown smooth function; y∈R is the output of the system; u∈R is the corresponding control input of the system; $\eta_{i}$ is the order of the system; $g_{i}$ is the unknown control gain parameter and satisfies $0<{\underline{g}}_{i}\leq g_{i}\leq {\bar{g}}_{i}$, where ${\underline{g}}_{i},\;{\bar{g}}_{i}$ are known parameters; and the desired trajectory $y_{d}$ and its derivative are continuous and bounded.

**Lemma** **1.** *For positive real numbers* $p,q,p\in \left(0,1\right),q\in \left(1,\infty \right)$*with a denominator and numerator, both are odd numbers and positive real numbers*$\rho ,\sigma ,\rho_{1},\rho_{2},\sigma_{1},\sigma_{2}$; *then, the following inequalities hold*:

(2) $$\begin{array}{l} -\rho \tilde{\theta }{\hat{\theta }}_{}^{p}\leq -\rho_{1}{\tilde{\theta }}_{}^{p+1}+\rho_{2}\theta_{}^{p+1}\\ -\sigma \tilde{\theta }{\hat{\theta }}_{}^{q}\leq -\sigma_{1}{\tilde{\theta }}_{}^{q+1}+\sigma_{2}\theta_{}^{q+1} \end{array}$$

*where*$\rho_{1},\rho_{2},\sigma_{1},\sigma_{2}$*are determined by*p,q,ρ,σ [39].

**Lemma** **2.** *For any constant where* x,y∈R*and*p,q*are odd, the following inequality holds*:

(3) $$x^{\xi }-y^{\xi }\leq \xi \left|x-y\right|\left(x^{\xi -1}+y^{\xi -1}\right)\leq \zeta \left|x-y\right|\left({\left(x-y\right)}^{\xi -1}+y^{\xi -1}\right)$$

*where*$\xi =\frac{q}{p}$*and*q>p>1, $\zeta =\xi \left(2^{\xi -2}+2\right)$.

**Proof.**  Assuming x≥y, for any constant, the following equation holds:

(4) $$\frac{x^{\xi }-y^{\xi }}{x-y}=\xi c^{\xi -1}$$

where c is an existent constant and satisfies y≤c≤x; therefore,

(5) $$\begin{array}{c} x^{\xi }-y^{\xi }=\xi c^{\xi -1}\left(x-y\right)\\ \;\;\;\leq \xi c^{\xi -1}\left|x-y\right| \end{array}$$

because y≤c≤x, then $c^{\xi -1}\leq \max\left\{x^{\xi -1},y^{\xi -1}\right\}\leq x^{\xi -1}+y^{\xi -1}$; therefore,

(6) $$x^{\xi }-y^{\xi }\leq \xi \left|x-y\right|\left(x^{\xi -1}+y^{\xi -1}\right)$$

On the other hand, based on ξ>1, for $x^{\xi -1},{\left(x-y\right)}^{\xi -1},y^{\xi -1}$, we have

(7) $$x^{\xi -1}\leq \left\{\begin{array}{c} {\left(x-y\right)}^{\xi -1}+y^{\xi -1},1<\xi <2\\ 2^{\xi -2}\left({\left(x-y\right)}^{\xi -1}+y^{\xi -1}\right),\xi \geq 2 \end{array}\right.$$

then, we choose

(8) $$x^{\xi -1}\leq \left(2^{\xi -2}+1\right)\left({\left(x-y\right)}^{\xi -1}+y^{\xi -1}\right)$$

therefore,

(9) $$x^{\xi -1}+y^{\xi -1}\leq \left(2^{\xi -2}+2\right)\left({\left(x-y\right)}^{\xi -1}+y^{\xi -1}\right)$$

Then,

(10) $$\xi \left|x-y\right|\left(x^{\xi -1}+y^{\xi -1}\right)\leq \zeta \left|x-y\right|\left({\left(x-y\right)}^{\xi -1}+y^{\xi -1}\right)$$

where $\zeta =\xi \left(2^{\xi -2}+2\right)$. □

## 3. Main Results

In this section, for the strict-feedback high-order nonlinear system, the neural network is used to identify the nonlinear system, and an adaptive algorithm is used to adjust the weight coefficient of the neural network. Based on fixed-time Lyapunov stability theory, a neural network adaptive tracker based on backstepping control strategy is designed so that the system state can track the preset trajectory. Theoretical proof and a numerical simulation are given.

**Figure 1:**
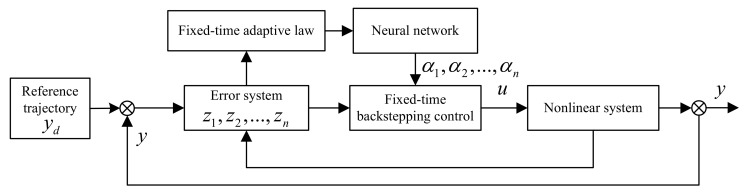
Block diagram of the closed-loop system.

The design block diagram of the closed-loop system is shown in Figure 1. For high-order nonlinear systems with strict-feedback form, a neural network adaptive controller is designed to make the system track a given target signal in finite time. The convergence time is independent of the initial condition to achieve fixed-time Lyapunov stability of the closed-loop error system. The controller design can be divided into the following N steps:

Step 1: First, for the system, the following variables are selected:

(11) $$z_{1}=x_{1}-y_{d}$$

the dynamics of $z_{1}$ can be obtained as

(12) $${\dot{z}}_{1}=g_{1}x_{2}^{\eta_{1}}+f_{1}\left(x_{1}\right)-{\dot{y}}_{d}$$

Moreover, we have

(13) $$f_{1}\left(x_{1}\right)=W_{1}^{T}\Psi \left(Z_{1}\right)+\varepsilon_{1}\left(Z_{1}\right)$$

where $\left|\varepsilon_{1}\left(x_{1}\right)\right|\leq \varepsilon_{1}$, we have

(14) $$z_{1}f_{1}\left(x_{1}\right)\leq \theta_{1}\left|z_{1}\right|\left\|\Psi \left(Z_{1}\right)\right\|+\left|z_{1}\right|\varepsilon_{1}$$

$\theta_{1}=\left\|W_{1}\right\|$ is defined, and the Lyapunov candidate functional is chosen as

(15) $$V_{1}=\frac{1}{2}z_{1}^{2}+\frac{1}{2\mu_{1}}{\tilde{\theta }}_{1}^{2}$$

where $\mu_{1}>0$ is positive constant, and ${\tilde{\theta }}_{1}={\hat{\theta }}_{1}-\theta_{1}$. Differentiating $V_{1}$ with respect to time t yields

(16) $${\dot{V}}_{1}\leq g_{1}z_{1}x_{2}^{\eta_{1}}+\theta_{1}\left|z_{1}\right|\left\|\Psi \left(Z_{1}\right)\right\|+\left|z_{1}\right|\varepsilon_{1}-z_{1}{\dot{y}}_{d}+\frac{1}{\mu_{1}}{\tilde{\theta }}_{1}{\dot{\hat{\theta }}}_{1}$$

The virtual control signal $\alpha_{1}$ is selected as

(17) $$\alpha_{1}=-{\underline{g}}_{1}^{-\frac{1}{\eta_{1}}}{\left(sign\left(z_{1}\right){\hat{\theta }}_{1}\left\|\Psi \left(Z_{1}\right)\right\|+\frac{z_{1}\varepsilon_{1}^{2}}{\left|z_{1}\right|\varepsilon_{1}+\eta_{1}}+sign\left(z_{1}\right)\left|{\dot{y}}_{d}\right|+\kappa_{1}z_{1}^{p}+\iota_{1}z_{1}^{q}\right)}^{\frac{1}{\theta_{1}}}$$

Then, based on Lemma 2, we have

(18) $${\dot{V}}_{1}\leq c_{1}{\bar{g}}_{1}\left|z_{1}\right|\left(\left|z_{2}^{\eta_{1}}\right|+\left|z_{2}\right|x_{2}^{\eta_{1}-1}\right)-\left|z_{1}\right|{\tilde{\theta }}_{1}\left\|\Psi \left(Z_{1}\right)\right\|+\delta_{1}+\frac{1}{\mu_{1}}{\tilde{\theta }}_{1}{\dot{\hat{\theta }}}_{1}-\kappa_{1}z_{1}^{p+1}-\iota_{1}z_{1}^{q+1}$$

where

(19) $$z_{2}=x_{2}-\alpha_{1}$$

then the adaptive law design as

(20) $${\dot{\hat{\theta }}}_{1}=\mu_{1}\left(\left|z_{1}\right|\left|\Psi_{1}\right|-\rho_{1}{\hat{\theta }}_{1}^{p}-\sigma_{1}{\hat{\theta }}_{1}^{q}\right)$$

based on Equation (18), we have

(21) $$\begin{array}{l} {\dot{V}}_{1}\leq c_{1}{\bar{g}}_{1}\left|z_{1}\right|\left(\left|z_{2}^{\eta_{1}}\right|+\left|z_{2}\right|x_{2}^{\eta_{1}-1}\right)-\left|z_{1}\right|{\tilde{\theta }}_{1}\left\|\Psi \left(Z_{1}\right)\right\|+\delta_{1}+{\tilde{\theta }}_{1}\left|z_{1}\right|\left|\Psi_{1}\right|\\ \;\;-\rho_{1}{\tilde{\theta }}_{1}{\hat{\theta }}_{1}^{p}-\sigma_{1}{\tilde{\theta }}_{1}{\hat{\theta }}_{1}^{q}-\kappa_{1}z_{1}^{p+1}-\iota_{1}z_{1}^{q+1} \end{array}$$

based on Lemma 1, we have

(22) $$\begin{array}{l} -\rho_{1}{\tilde{\theta }}_{1}{\hat{\theta }}_{1}^{p}\leq -\varsigma_{1}{\tilde{\theta }}_{1}^{p+1}+\upsilon_{1}\theta_{1}^{p+1}\\ -\sigma_{1}{\tilde{\theta }}_{1}{\hat{\theta }}_{1}^{q}\leq -\omega_{1}{\tilde{\theta }}_{1}^{q+1}+\vartheta_{1}\theta_{1}^{q+1} \end{array}$$

then

(23) $${\dot{V}}_{1}\leq c_{1}{\bar{g}}_{1}\left|z_{1}\right|\left(\left|z_{2}^{\theta_{1}}\right|+\left|z_{2}\right|x_{2}^{\theta_{1}-1}\right)+\delta_{1}-\varsigma_{1}{\tilde{\theta }}_{1}^{p+1}+\upsilon_{1}\theta_{1}^{p+1}-\omega_{1}{\tilde{\theta }}_{1}^{q+1}+\vartheta_{1}\theta_{1}^{q+1}-\kappa_{1}z_{1}^{p+1}-\iota_{1}z_{1}^{q+1}$$

Step i: the tracking error can be described as

(24) $$z_{i}=x_{i}-\alpha_{i-1}$$

Based on dynamics and tracking error, the dynamics of $z_{i}$ can be obtained as

(25) $${\dot{z}}_{i}=g_{i}x_{i+1}^{\theta_{i}}+f_{i}\left(x_{i}\right)-{\dot{\alpha }}_{i-1}$$

Moreover, we have

(26) $$f_{i}\left(x_{i}\right)-{\dot{\alpha }}_{i-1}=W_{i}^{T}\Psi \left(Z_{i}\right)+\varepsilon_{i}\left(Z_{i}\right)$$

where $\left|\varepsilon_{i}\left(x_{i}\right)\right|\leq \varepsilon_{i}$; we have

(27) $$z_{i}\left(f_{i}\left({\bar{x}}_{i}\right)-{\dot{\alpha }}_{i-1}\right)\leq \theta_{i}\left|z_{i}\right|\left\|\Psi \left(Z_{i}\right)\right\|+\left|z_{i}\right|\varepsilon_{i}$$

$\theta_{i}=\left\|W_{i}\right\|$ is defined, and the Lyapunov candidate functional is chosen as

(28) $$V_{i}=\frac{1}{2}z_{i}^{2}+\frac{1}{2\mu_{i}}{\tilde{\theta }}_{i}^{2}$$

where $\mu_{i}>0$ is positive constant and ${\tilde{\theta }}_{i}={\hat{\theta }}_{i}-\theta_{i}$. Differentiating $V_{i}$ with respect to time t, yields

(29) $${\dot{V}}_{i}\leq g_{i}z_{i}x_{i+1}^{\theta_{i}}+\theta_{i}\left|z_{i}\right|\left\|\Psi \left(Z_{i}\right)\right\|+\left|z_{i}\right|\varepsilon_{i}+\frac{1}{\mu_{i}}{\tilde{\theta }}_{i}{\dot{\hat{\theta }}}_{i}$$

The virtual control signal $\alpha_{i}$ is designed as

(30) $$\alpha_{i}=-{\underline{g}}_{i}^{-\frac{1}{\eta_{i}}}{\left(\begin{array}{l} c_{i-1}sign\left(z_{i}\right){\bar{g}}_{i-1}\left|z_{i-1}\right|\left(z_{i}^{\eta_{i-1}-1}+x_{i}^{\eta_{i-1}-1}\right)+sign\left(z_{i}\right){\hat{\theta }}_{i}\left\|\Psi \left(Z_{i}\right)\right\|\\ +\frac{z_{i}\varepsilon_{i}^{2}}{\left|z_{i}\right|\varepsilon_{i}+\delta_{i}}+\kappa_{i}z_{i}^{p}+\iota_{i}z_{i}^{q} \end{array}\right)}^{\frac{1}{\theta_{i}}}$$

then, based on Lemma 2, we have

(31) $$\begin{array}{l} {\dot{V}}_{i}\leq -c_{i-1}{\bar{g}}_{i-1}\left|z_{i-1}\right|\left(\left|z_{i}^{\eta_{i-1}}\right|+\left|z_{i}\right|x_{i}^{\eta_{i-1}-1}\right)+c_{i}{\bar{g}}_{i}\left|z_{i}\right|\left(\left|z_{i+1}^{\eta_{i}}\right|+\left|z_{i+1}\right|x_{i+1}^{\eta_{i}-1}\right)\\ \;\;-\left|z_{i}\right|{\tilde{\theta }}_{i}\left\|\Psi \left(Z_{i}\right)\right\|+\eta_{i}+\frac{1}{\mu_{i}}{\tilde{\theta }}_{i}{\dot{\hat{\theta }}}_{i}-\kappa_{i}z_{i}^{p+1}-\iota_{i}z_{i}^{q+1} \end{array}$$

where

(32) $$z_{i+1}=x_{i+1}-\alpha_{i}$$

Then, the adaptive law design as

(33) $${\hat{\theta }}_{i}=\mu_{i}\left(\left|z_{i}\right|\left|\Psi_{i}\right|-\rho_{i}{\hat{\theta }}_{i}^{p}-\sigma_{i}{\hat{\theta }}_{i}^{q}\right)$$

based on Equation (31), we have

(34) $$\begin{array}{l} {\dot{V}}_{i}\leq -c_{i-1}{\bar{g}}_{i-1}\left|z_{i-1}\right|\left(\left|z_{i}^{\eta_{i-1}}\right|+\left|z_{i}\right|x_{i}^{\eta_{i-1}-1}\right)+c_{i}{\bar{g}}_{i}\left|z_{i}\right|\left(\left|z_{i+1}^{\eta_{i}}\right|+\left|z_{i+1}\right|x_{i+1}^{\eta_{i}-1}\right)-\left|z_{i}\right|{\tilde{\theta }}_{i}\left\|\Psi \left(Z_{i}\right)\right\|+\eta_{i}\\ \;\;+{\tilde{\theta }}_{i}\left|z_{i}\right|\left|\Psi_{i}\right|-\rho_{i}{\tilde{\theta }}_{i}{\hat{\theta }}_{i}^{p}-\sigma_{i}{\tilde{\theta }}_{i}{\hat{\theta }}_{i}^{q}-\kappa_{i}z_{i}^{p+1}-\iota_{i}z_{i}^{q+1} \end{array}$$

based on Lemma 1, we have

(35) $$\begin{array}{l} -\rho_{i}{\tilde{\theta }}_{i}{\hat{\theta }}_{i}^{p}\leq -\varsigma_{i}{\tilde{\theta }}_{i}^{p+1}+\upsilon_{i}\theta_{i}^{p+1}\\ -\sigma_{i}{\tilde{\theta }}_{i}{\hat{\theta }}_{i}^{q}\leq -\omega_{i}{\tilde{\theta }}_{i}^{q+1}+\vartheta_{i}\theta_{i}^{q+1} \end{array}$$

then

(36) $$\begin{array}{l} {\dot{V}}_{i}\leq -c_{i-1}{\bar{g}}_{i-1}\left|z_{i-1}\right|\left(\left|z_{i}^{\eta_{i-1}}\right|+\left|z_{i}\right|x_{i}^{\eta_{i-1}-1}\right)+c_{i}{\bar{g}}_{i}\left|z_{i}\right|\left(\left|z_{i+1}^{\eta_{i}}\right|+\left|z_{i+1}\right|x_{i+1}^{\eta_{i}-1}\right)+\delta_{i}-\varsigma_{i}{\tilde{\theta }}_{i}^{p+1}+\upsilon_{i}\theta_{i}^{p+1}\\ \;\;-\omega_{i}{\tilde{\theta }}_{i}^{q+1}+\vartheta_{i}\theta_{i}^{q+1}-\kappa_{i}z_{i}^{p+1}-\iota_{i}z_{i}^{q+1} \end{array}$$

Step *n*: the time derivative of $z_{n}$ can be described as

(37) $$z_{n}=x_{n}-\alpha_{n-1}$$

Based on dynamics and tracking error, the dynamics of $z_{n}$ can be obtained as

(38) $${\dot{z}}_{n}=g_{n}u^{\eta_{n}}+f_{n}\left({\bar{x}}_{n}\right)-{\dot{\alpha }}_{n-1}$$

Moreover, we have

(39) $$f_{n}\left({\bar{x}}_{n}\right)-{\dot{\alpha }}_{n-1}=W_{n}^{T}\Psi \left(Z_{n}\right)+\varepsilon_{n}\left(Z_{n}\right)$$

where $\left|\varepsilon_{n}\left(x_{n}\right)\right|\leq \varepsilon_{n}$, we have

(40) $$z_{n}\left(f_{n}\left({\bar{x}}_{n}\right)-{\dot{\alpha }}_{n-1}\right)\leq \theta_{n}\left|z_{n}\right|\left\|\Psi \left(Z_{n}\right)\right\|+\left|z_{n}\right|\varepsilon_{n}$$

$\theta_{n}=\left\|W_{n}\right\|$ is defined, and the Lyapunov candidate functional is chosen as

(41) $$V_{n}=\frac{1}{2}z_{n}^{2}+\frac{1}{2\mu_{n}}{\tilde{\theta }}_{n}^{2}$$

where $\mu_{n}>0$ is positive constant and ${\tilde{\theta }}_{n}={\hat{\theta }}_{n}-\theta_{n}$. Differentiating $V_{n}$ with respect to time t yields

(42) $${\dot{V}}_{n}\leq g_{n}z_{n}u^{\theta_{n}}+\theta_{n}\left|z_{n}\right|\left\|\Psi \left(Z_{n}\right)\right\|+\left|z_{n}\right|\varepsilon_{n}+\frac{1}{\mu_{n}}{\tilde{\theta }}_{n}{\dot{\hat{\theta }}}_{n}$$

The actual control is designed as

(43) $$u=-{\underline{g}}_{n}^{-\frac{1}{\eta_{n}}}{\left(\begin{array}{l} c_{n-1}sign\left(z_{n}\right){\bar{g}}_{n-1}\left|z_{n-1}\right|\left(z_{n}^{\theta_{n-1}-1}+x_{n}^{\theta_{n-1}-1}\right)+sign\left(z_{n}\right){\hat{\theta }}_{n}\left\|\Psi \left(Z_{n}\right)\right\|\\ +\frac{z_{n}\varepsilon_{n}^{2}}{\left|z_{n}\right|\varepsilon_{n}+\delta_{n}}+\kappa_{n}z_{n}^{p}+\iota_{n}z_{n}^{q} \end{array}\right)}^{\frac{1}{\eta_{n}}}$$

then, based on Lemma 2, we have

(44) $$\begin{array}{l} {\dot{V}}_{n}\leq -c_{n-1}{\bar{g}}_{n-1}\left|z_{n-1}\right|\left(\left(\left|z_{n}^{\eta_{n-1}}\right|+\left|z_{n}\right|x_{n}^{\eta_{n-1}-1}\right)\right)-\left|z_{n}\right|{\tilde{\theta }}_{n}\left\|\Psi \left(Z_{n}\right)\right\|+\eta_{n}\\ \;\;+\frac{1}{\mu_{n}}{\tilde{\theta }}_{n}{\dot{\hat{\theta }}}_{n}-\kappa_{n}z_{n}^{p+1}-\iota_{n}z_{n}^{q+1} \end{array}$$

then, the adaptive law design as

(45) $${\hat{\theta }}_{n}=\mu_{n}\left(\left|z_{n}\right|\left|\Psi_{n}\right|-\rho_{n}{\hat{\theta }}_{n}^{p}-\sigma_{n}{\hat{\theta }}_{n}^{q}\right)$$

based on Equation (20), we have

(46) $${\dot{V}}_{n}\leq -c_{n-1}{\bar{g}}_{n-1}\left|z_{n-1}\right|\left(\left(\left|z_{n}^{\eta_{n-1}}\right|+\left|z_{n}\right|x_{n}^{\eta_{n-1}-1}\right)\right)+\eta_{n}-\kappa_{n}z_{n}^{p+1}-\iota_{n}z_{n}^{q+1}-\rho_{n}{\tilde{\theta }}_{n}{\hat{\theta }}_{n}^{p}-\sigma_{n}{\tilde{\theta }}_{n}{\hat{\theta }}_{n}^{q}$$

based on Lemma 1, we have

(47) $$\begin{array}{l} -\rho_{n}{\tilde{\theta }}_{n}{\hat{\theta }}_{n}^{p}\leq -\varsigma_{n}{\tilde{\theta }}_{n}^{p+1}+\upsilon_{n}\theta_{n}^{p+1}\\ -\sigma_{n}{\tilde{\theta }}_{n}{\hat{\theta }}_{n}^{q}\leq -\omega_{n}{\tilde{\theta }}_{n}^{q+1}+\vartheta_{n}\theta_{n}^{q+1} \end{array}$$

then

(48) $$\begin{array}{l} {\dot{V}}_{n}\leq -c_{n-1}{\bar{g}}_{n-1}\left|z_{n-1}\right|\left|z_{n}^{\eta_{n}-1}\right|+\delta_{n}-\kappa_{n}z_{n}^{p+1}-\iota_{n}z_{n}^{q+1}\\ \;\;-\varsigma_{n}{\tilde{\theta }}_{n}^{p+1}+\upsilon_{n}\theta_{n}^{p+1}-\omega_{n}{\tilde{\theta }}_{n}^{q+1}+\vartheta_{n}\theta_{n}^{q+1} \end{array}$$

**Theorem** **1.** *For the strict-feedback high-order nonlinear system with unknown nonlinearity (1), based on the feasible virtual control signal (17), (30), actual controller (43), and adaptive function (20) (33), the error system is fixed-time Lyapunov stable, and the convergence time is independent of the initial condition*.

**Proof.**  Based on Lyapunov candidate functional (15), (28), (41), the Lyapunov candidate functional is chosen.

(49) $$V=\sum_{j=1}^{n}V_{i}$$

The virtual control signal is chosen as (17), (30), and the fixed-time adaptive function is chosen as (20), (33); the controller is designed as (43) according to the fixed-time Lyapunov stability theory. Based on fixed-time adaptive neural network control and backstepping technology, and taking the trajectory along the system, we have

(50) $$\begin{array}{l} \dot{V}\leq -\sum_{j=1}^{n}\kappa_{j}z_{j}^{p+1}-\sum_{j=1}^{n}\iota_{j}z_{j}^{q+1}-\sum_{j=1}^{n}\varsigma_{j}{\tilde{\theta }}_{j}^{p+1}-\sum_{j=1}^{n}\omega_{j}{\tilde{\theta }}_{j}^{q+1}\\ \;+\sum_{j=1}^{n}\eta_{j}+\sum_{j=1}^{n}\upsilon_{j}\theta_{j}^{p+1}+\sum_{j=1}^{n}\vartheta_{j}\theta_{j}^{q+1}\\ \;\leq -aV^{\frac{p+1}{2}}-bV^{\frac{q+1}{2}}+c \end{array}$$

where

(51) $$\begin{array}{l} a=\frac{\min\left\{\kappa_{j\in N},\varsigma_{j\in N}\right\}}{{\left(\max\left\{\frac{1}{2},\frac{1}{2\mu_{j\in N}}\right\}\right)}^{\frac{p+1}{2}}},b=\frac{{\left(2n\right)}^{\frac{1-q}{2}}\min\left\{\iota_{j\in N},\;\omega_{j\in N}\right\}}{{\left(\max\left\{\frac{1}{2},\frac{1}{2\mu_{j\in N}}\right\}\right)}^{\frac{q+1}{2}}}\\ c=\sum_{j=1}^{n}\delta_{j}+\sum_{j=1}^{n}\upsilon_{j}\theta_{j}^{p+1}+\sum_{j=1}^{n}\vartheta_{j}\theta_{j}^{q+1} \end{array}$$

Therefore, according to the lemma in [39], all closed-loop signals possess fixed-time Lyapunov stability. □

**Figure 2:**
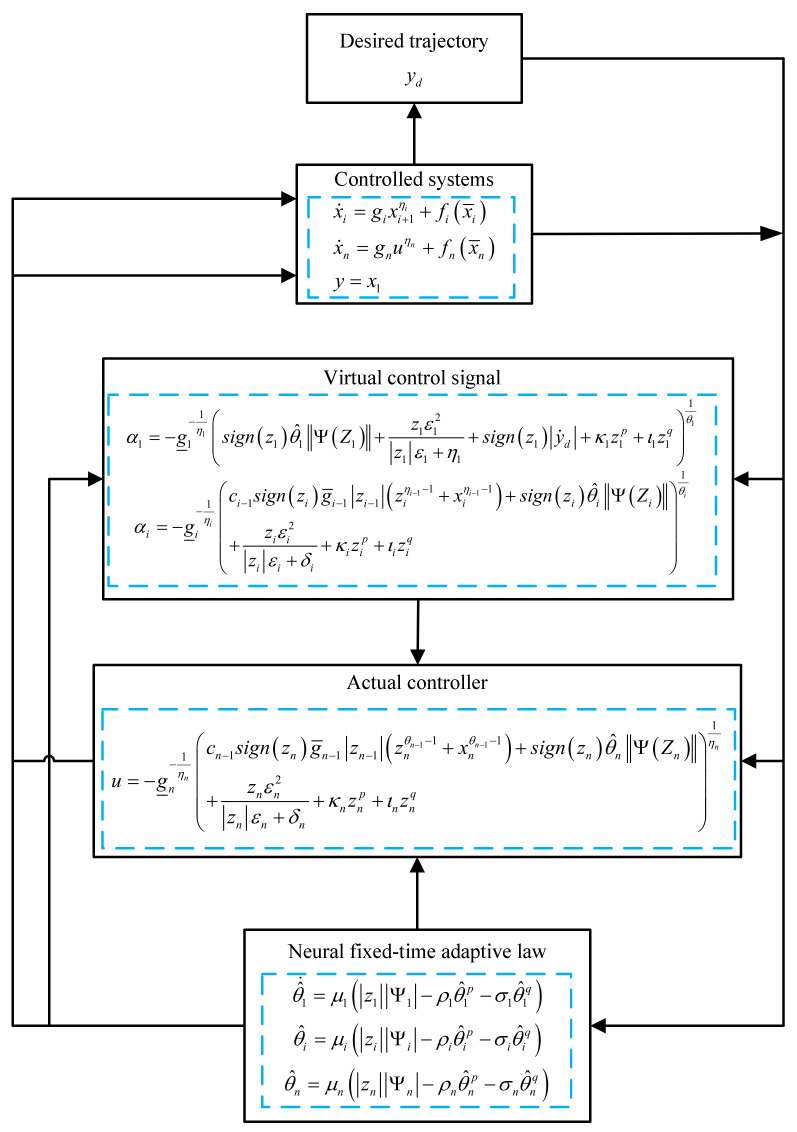
Design procedure.

The design details are summarized in Figure 2 to show the procedure of the control process.

## 4. Numerical Examples

In this paper, the feasibility and effectiveness of the algorithm are verified by numerical simulation. A strict-feedback high-order system is considered as follows:

(52) $$\left\{\begin{array}{l} {\dot{x}}_{1}=g_{1}x_{2}^{\eta_{1}}+f_{1}\left(x_{1}\right)\\ {\dot{x}}_{2}=g_{2}u^{\eta_{2}}+f_{2}\left(x_{1},x_{2}\right)\\ y=x_{1} \end{array}\right.$$

where the function $f_{1}\left(x_{1}\right)=x_{1}\left(t\right)+\sin\left(0.1x_{1}\left(t\right)\right)$, $f_{2}\left(x_{1},x_{2}\right)=x_{2}\left(t\right)$, $g_{1}=1$, $g_{2}=1$, $\eta_{1}=\frac{5}{3}$, $\eta_{2}=\frac{7}{5}$, and the control input under the adaptive law is designed

(53) $${\dot{\hat{\theta }}}_{1}=0.01\left(\left|z_{1}\right|\left|\Psi_{1}\right|-0.1{\hat{\theta }}_{1}^{\frac{5}{3}}-0.1{\hat{\theta }}_{1}^{\frac{1}{3}}\right)$$

(54) $${\dot{\hat{\theta }}}_{2}=0.01\left(\left|z_{2}\right|\left|\Psi_{2}\right|-0.1{\hat{\theta }}_{2}^{\frac{5}{3}}-0.1{\hat{\theta }}_{2}^{\frac{1}{3}}\right)$$

the control input is designed as

(55) $$u\left(t\right)={\left(\begin{array}{l} -5sign\left(z_{2}\left(t\right)\right)*\left|z_{1}\left(t\right)\right|*\left(\left|z_{2}\left(t\right)\right|+{\left|x_{2}\left(t\right)\right|}^{\frac{2}{5}}\right)+sign\left(z_{2}\left(t\right)\right){\hat{\theta }}_{2}\Psi_{2}\left(Z_{2}\right)\\ +\frac{0.01z_{2}}{0.1\left|z_{2}\left(t\right)\right|+0.1}+5z_{2}{\left(t\right)}^{\frac{1}{3}}+5z_{2}{\left(t\right)}^{\frac{5}{3}} \end{array}\right)}^{\frac{5}{7}}$$

The desired reference signal is $y_{d}=\sin\left(t\right)$. The initial condition is selected as $x_{1}\left(0\right)=1,x_{2}\left(0\right)=1,{\hat{\theta }}_{1}\left(0\right)=1,{\hat{\theta }}_{2}\left(0\right)=1$. The neural network consists of seven nodes, centers $c=\left[-3,-2,-1,0,1,2,3\right]$, and widths b=1.

**Figure 3:**
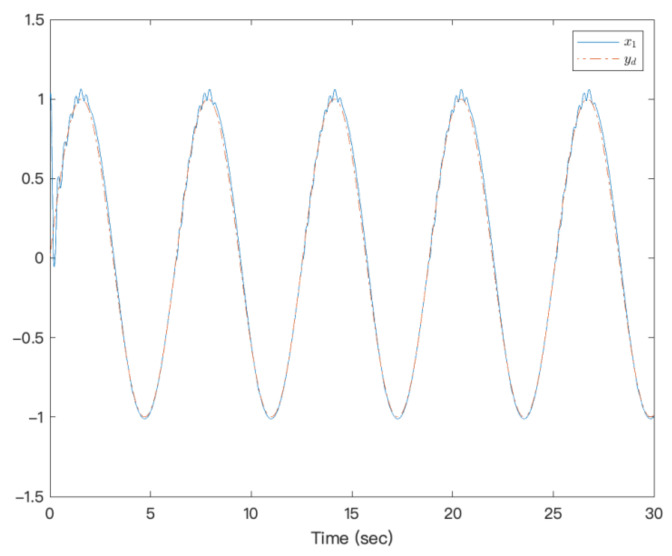
Trajectories of $x_{1}$ and $y_{d}$ of a strict-feedback high-order nonlinear system.

**Figure 4:**
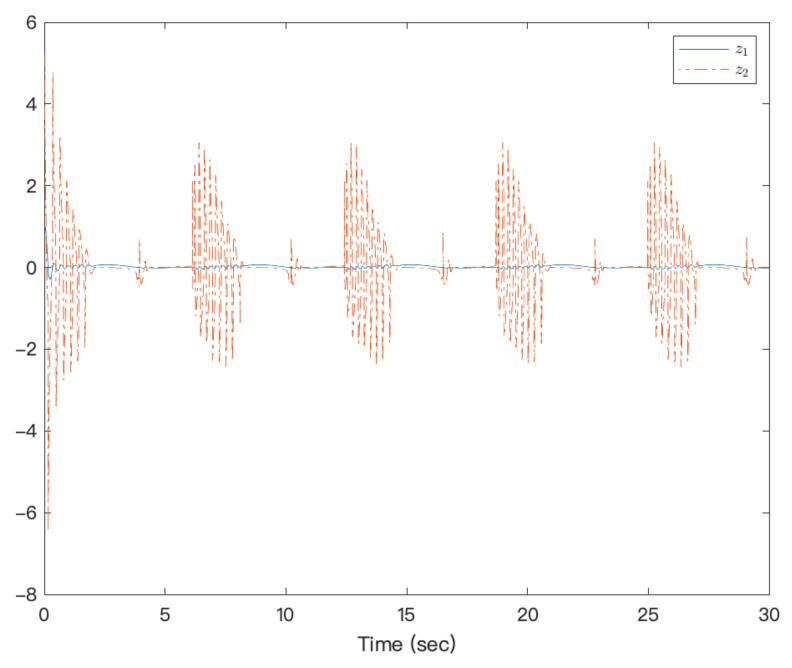
Trajectories of error states of a strict-feedback high-order nonlinear system.

**Figure 5:**
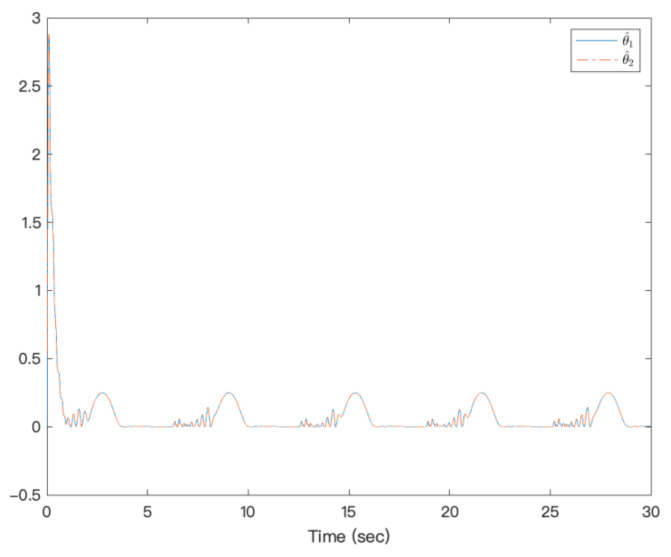
Trajectories of adaptive functions of a strict-feedback high-order nonlinear system.

**Figure 6:**
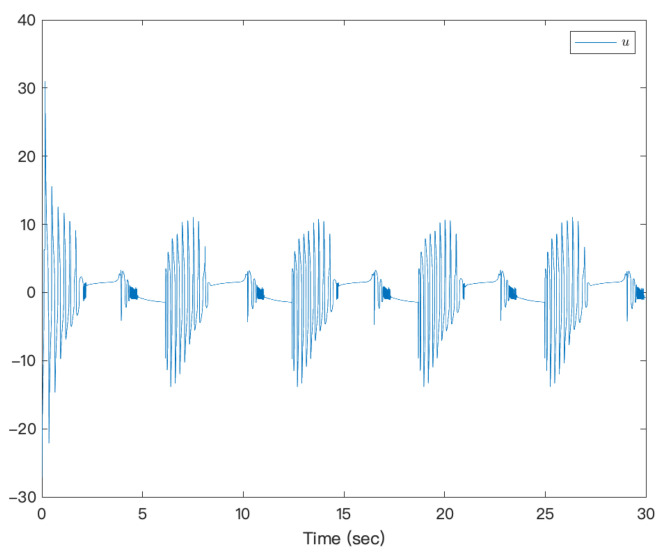
Trajectories of system input of a strict-feedback high-order nonlinear system.

Figure 3 shows that under the action of the neural network adaptive controller, the state of the controlled system state can track the preset trajectory in finite time. Figure 4 shows the state trajectory of the error system. It can be seen from the figure that under the action of the controller, the error system achieves fixed-time Lyapunov stability. The adaptive function curve is shown in Figure 5. For fixed-time control, $\alpha_{1}$ is designed by $f_{1}\left(x_{1}\right)$, which is approximated by NNs, but its derivative is not easy to approximate; therefore, $f_{2}\left({\bar{x}}_{2}\right)-{\dot{\alpha }}_{1}$ is not easy to approximate, and the amplitude is inevitable. Figure 6 shows that the system’s controllers are bounded. It can be seen from the figures that the designed method is effective.

## 5. Conclusions

In this paper, based on backstepping adaptive control technology, a neural network is used to approximate some unknown signals in a system. Combined with Lyapunov stability theorem and fixed time stability, an effective adaptive control scheme is designed. A class of strict-feedback high-order systems is further studied. The main contributions of this paper are as follows: the fixed-time control problem of strict-feedback high-order nonlinear systems is solved; the Lyapunov function is designed for each subsystem; at the same time, combined with adaptive backstepping technology, an adaptive neural network fixed-time controller is designed. The tracking error converges in finite time through stability analysis, and the convergence time does not relay on the initial condition. The most popular controller is designed in a linear control strategy, which controls the state’s exponential stability. At present, the adaptive neural network control method based on backstepping has some limitations, and many problems need to be further studied and solved. In the finite-time adaptive control method for the multi-agent system, the finite time obtained by most finite-time control strategies often depends on the initial conditions of the system. Therefore, a finite-time control scheme independent of the initial value for a multi-agent system must be designed.

## Data Availability

Not applicable.
